# Risk factors of a reduced response to ranibizumab treatment for neovascular age-related macular degeneration – evaluation in a clinical setting

**DOI:** 10.1186/1471-2415-13-84

**Published:** 2013-12-20

**Authors:** Christina Korb, Isabella Zwiener, Katrin Lorenz, Alireza Mirshahi, Norbert Pfeiffer, Bernhard Stoffelns

**Affiliations:** 1 Department of Ophthalmology, University Medical Centre, Johannes Gutenberg-University, Langenbeckstr.1, Mainz 55131, Germany; 2 Institute of Medical Biostatistics, Epidemiology and Informatics (IMBEI), Johannes Gutenberg-University, Mainz, Germany

**Keywords:** Ranibizumab, Lucentis, Age-related macular degeneration, Response to treatment

## Abstract

**Background:**

To identify risk factors for being a “reduced responder” to ranibizumab treatment in a clinical setting in patients with neovascular age-related macular degeneration.

**Methods:**

This retrospective study included 165 eyes of 165 consecutive patients with choroidal neovascularisation secondary to neovascular, age-related macular degeneration. Eyes were treated with three intravitreal injections of ranibizumab, followed by PRN (pro re nata) dosing thereafter. All patients were reevaluated every four weeks and then followed for six months. Reduced responders were defined as patients with a loss in visual acuity of at least 1 visual acuity line at the last follow-up and/or persistent intraretinal or subretinal fluid or detectable choroidal neovascularisation at the last follow-up, compared to baseline.

**Results:**

Overall, 58 out of 165 eyes (35.2%) were considered to be reduced responders to treatment at the end of follow-up. The initial CNV size at baseline was correlated with the risk of being a reduced responder at the end of follow-up (p = 0.017).

**Conclusion:**

We identified the initial lesion size as a predictor for a reduced response to treatment in this study. Patients with a large initial lesion size should be thoroughly informed about the possible poorer response to the intravitreal treatment.

## Background

Ranibizumab is a humanised antigen-binding fragment (Fab) that targets all isoforms of vascular endothelial growth factor A (VEGF-A) and is approved by the Food and Drug Administration for the treatment of patients with neovascular age-related macular degeneration (AMD), as well as diabetic macular oedema and macular oedema following retinal vein occlusion. Randomised phase-III clinical trials (*M*inimally Classic/Occult Trial of the *A*nti-VEGF Antibody *R*anibizumab *I*n the Treatment of *N*eovascular *A*ge-Related Macular Degeneration [Marina] and *An*ti-VEGF Antibody for the Treatment of Predominantly Classic *Chor*oidal Neovascularisation in Age-Related Macular Degeneration [ANCHOR]) showed a reduction in retinal thickness and maintained visual acuity gains with monthly intravitreal injections of 0.3 and 0.5 mg of ranibizumab for treating minimally classic, occult and predominantly classic CNV secondary to AMD [1,2]. The “*Pr*ospective *O*ptical Coherence Tomography (OCT) Imaging of Patients with *N*eovascular Age-Related Macular Degeneration (AMD) *T*reated with intra*O*cular Ranibizumab [PrONTO]” trial explored an alternate dosing strategy of intravitreal ranibizumab for all types of subfoveal CNV secondary to AMD. Patients underwent three consecutive monthly injections followed by PRN (pro re nata) dosing thereafter [3]. After twelve months, visual acuity improved 15 or more letters in 35% of patients [3].

However, publications about the limited response to anti-VEGF treatment are rare; the “reduced responder” poses challenges to clinicians, and there is no general consensus on how a reduced response is defined. There are very few current predictors of visual outcome.

In this retrospective study, the treatment of neovascular macular degeneration consisted of three consecutive injections of ranibizumab, followed by PRN dosing thereafter in a clinical setting. In a clinical setting, we investigated the determinants of a reduced response to treatment, defined as patients who revealed a reduction in visual acuity of at least 1 visual acuity line and/or persistent or recurrent retinal fluid or choroidal neovascularisation after six months of treatment, compared to baseline, after primary intravitreal ranibizumab therapy for choroidal neovascular lesions secondary to AMD.

## Methods

This retrospective data analysis was conducted at the Department of Ophthalmology, University Medical Centre of Johannes Gutenberg-University of Mainz, Germany. In total, 165 eyes of 165 consecutive patients with choroidal neovascularisation secondary to neovascular age-related macular degeneration who were treated within a nine-month time frame and completed the six-month follow-up were included in the study. Eyes were treated with three monthly injections of ranibizumab (Lucentis; Novartis, Nürnberg, Germany; 0.5 mg/0.05 ml) followed by PRN dosing. Retreatments occurred in case of progression (vision loss of at least 1 visual acuity line, increase in macular oedema of >100 μm, persistent leakage in fluorescein angiography, clinically detectable new haemorrhages). All patients were reevaluated every four weeks and then followed for six months. Approval from the local ethics committee was sought and waived due to the study’s retrospective nature. The study followed the tenets of the Declaration of Helsinki.

All lesion types were included in the study. No patient had undergone prior treatment or received additional therapy for neovascular AMD during follow-up.

Eyes received treatment after a complete ocular examination, including a best corrected distance visual acuity test (Snellen chart, BCVA was converted into logarithm of the minimum angle of resolution (logMAR) for statistical analysis), slit lamp examination, Goldmann applanation tonometry, binocular ophthalmoscopy, fundus colour photography, optical coherence tomography (fast macular thickness acquisition protocol, Stratus OCT, Zeiss Jena GmbH, Jena, Germany), fluorescein angiography (FA, HRA II, Heidelberg Engineering, Heidelberg, Germany), and indocyanine green angiography (ICGA, HRA II, Heidelberg Engineering, Heidelberg, Germany). The size of the CNV in the angiograms (greatest linear dimension, GLD) was measured on the middle phase fluorescein angiogram to exclude leakage during any later phases. Whenever ICG was also performed, those images were used to identify feeder vessels and to detect choroidal neovascularisation. Patients were re-scheduled for follow-up visits every 4 weeks. BCVA, slit lamp and binocular examinations took place at monthly intervals, and OCT, FA and ICGA at least every three months.

Before therapy, written informed consent was obtained from all patients after the potential risks and benefits of the intravitreal injections had been explained in detail.

All patients underwent intravitreal injections of ranibizumab via pars plana under topical anaesthesia under strict aseptic conditions.

Reduced responders were defined as follows:

– loss in visual acuity ≥1 visual acuity line at the last follow-up compared to baseline and/or

– persistent or recurrent intraretinal or subretinal fluid or detectable choroidal neovascularisation at the last follow-up.

As we refer to a “reduced response” in our paper and not to “non-response”, we applied the quite stringent criteria of any vision loss compared to baseline.

Statistical analysis was performed using SPSS statistical software (version 18.0, SPSS Inc., Chicago, IL, USA).

A logistic regression model was used to assess the influence of different variables on response to ranibizumab therapy. Our primary question was whether the CNV’s initial size would reveal an influence on the likelihood of a reduced response at the end of follow-up. Furthermore, we assessed the influence of the presence of an initial pigment epithelial detachment, initial central retinal thickness, lesion type, patient age and time elapsed from first examination in the clinic until time of the first injection.

The level of statistical significance was fixed at α = 0.05 for the primary hypothesis. All other p-values were considered to be explorative.

## Results

A total of 165 eyes of 165 patients were enrolled in this study, of which 98 patients were female. Patient age ranged from 56 to 94 years (mean 78 years). All of the patients completed the six-month follow-up. No major ocular or systemic adverse events were observed in the follow-up period.

**Figure 1:**
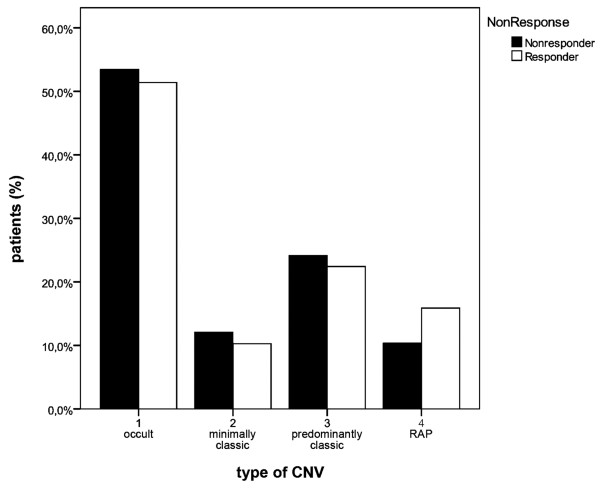
**Proportion of reduced responders (black) and responders (white) in the various CNV types (occult, minimally classic, predominantly classic and RAP).** The type of lesion was not related to the risk of a reduced response to treatment.

The lesions were classified as occult with no classic CNV in 86 eyes (52.1%), minimally classic CNV in 18 eyes (10.9%), predominantly classic CNV in 38 eyes (23%) and RAP lesions in the remaining 23 eyes (13.9%); see Figure 1. At baseline, 20 eyes (12.1%) presented a pigment epithelium detachment. The lesion location was subfoveal in 130 eyes (78.8%), parafoveal in 26 eyes (15.8%), and extrafoveal in 9 eyes (5.5%).

During the six-month follow-up, the majority of eyes (135 eyes, 81.8%) received the three consecutive injections in the first three months of treatment and needed no retreatment during follow-up, while 29 eyes (17.6%) received four injections and in one eye (0.6%), five injections of ranibizumab.

**Figure 2:**
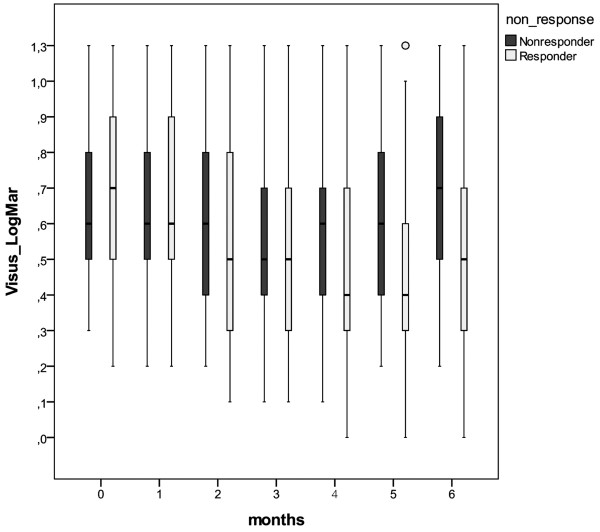
Change in mean visual acuity (LogMar) of reduced responders (black) and responders (white) at baseline (month 0) to the end of follow-up (month 6).

At baseline, the mean logMAR BCVA was 0.70 ± 0.30 (mean ± SD); the mean visual acuity improved to 0.55 ± 0.30 after the three consecutive injections and was 0.62 ± 0.33 at the end of follow-up; see Figure 2.

**Figure 3:**
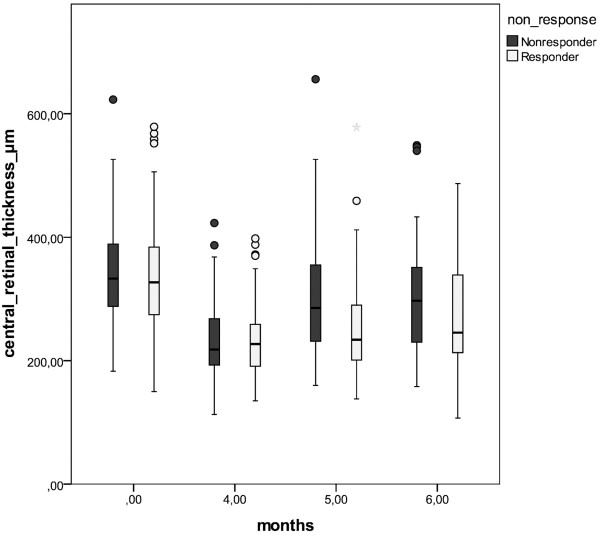
Change in mean retinal thickness (μm) of reduced responders (black) and responders (white) at baseline (month 0) and after the first three consecutive injections (month 4) to the end of follow-up (month 6).

The initial foveal thickness was 339 ± 84 μm (mean ± SD). After the three monthly consecutive injections of ranibizumab, this value decreased to 234 ± 59 μm and measured 280 ± 89 μm at the end of follow-up; see Figure 3.

**Figure 4:**
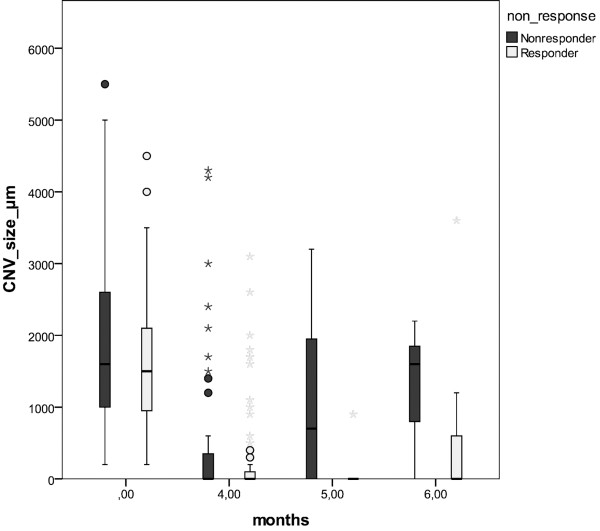
Change in the mean CNV size (μm) of reduced responders (black) and responders (white) at baseline (month 0) and after the first three consecutive injections (month 4), until the end of follow-up (month 6).

At baseline, the mean CNV size (GLD, greatest linear dimension) was 1736 ± 1093 μm (mean ± SD) initially; 338 ± 777 μm, after the first three injections; and 750 ± 885 μm, at the end of follow-up; see Figure 4.

One patient (0.6%) had lost three or more lines of vision at the end of follow-up, while 29 patients (17.6%) had gained three or more lines at the end of follow-up compared to baseline.

According to the criteria listed above, 58 out of 165 eyes (35.2%) were considered to be reduced responders to the treatment at the end of follow-up, presenting either a reduction in visual acuity compared to baseline and/or persistent intraretinal or subretinal fluid or persistent or recurrent choroidal neovascularisation.

We related the initial CNV size at baseline to the risk of being a reduced responder at the end of follow-up; see Figure 1. The OR (odds ratio) was 0.964 per 100 μm increase of the initial CNV size (GLD) (95% CI, 0.936-0.993, p = 0.017); see Table 1.

**Table 1:** Univariable logistic regression models were used to assess the influence of different variables (initial CNV size (GLD), initial PED, initial central retinal thickness, initial type of lesion, age of patient, time between first consultation to first injection) on response to ranibizumab therapy

	**OR**	**SD (95% CI)**	**p**
initial CNV size (GLD)	0.964	0.936-0.993	0.017
initial PED	1.728	0.595-5.024	0.315
initial central retinal thickness	0.998	0.994-1.002	0.362
initial type of lesion			
minimally classic vs. occult	0.886	0.312-2.518	0.820
predominantly classic vs. occult	0.966	0.437-2.135	0.932
RAP vs. occult	1.597	0.570-4.471	0.373
age of patient (years)	0.986	0.943-1.030	0.528
time between first consultation to first injection (days)	0.995	0.981-1.009	0.452

Shown are odds ratios (OR), standard deviations (SD) and p-values.

Table 1 shows that none of the initial presence of a pigment epithelium detachment (OR 1.728, 95% CI, 0.595-5.024, p = 0.315), the central retinal thickness at baseline (OR 0.998, 95% CI, 0.994-1.002, p = 0.362), the lesion type (OR 0.887, 0.966, 1.597 for minimally classic, predominantly classic, RAP vs. occult, p = 0.820, p = 0.932, p = 0.373, respectively), the patient age (OR 0.986, 95% CI, 0.943-1.030, p = 0.513), or the time between the first patient consultation and the first injection (OR 0.995, 95% CI, 0.981-1.009, p = 0.425) were related to the likelihood of a reduced response to treatment.

## Discussion

We related the responsiveness to intravitreal ranibizumab in our study to the initial lesion size at baseline. Lux et al. showed that the efficiency of intravitreal bevacizumab in patients with neovascular AMD depended on the initial lesion size [4]. This outcome corresponds with the findings of the TAP and VIP trials, which also reported the initial lesion size as an important predictor of the magnitude of treatment benefit with verteporfin therapy in occult, with no classic or minimally classic lesion, compositions [5].

Before the use of ranibizumab, treatments, such as verteporfin photodynamic therapy (Visudyne, Novartis Pharmaceuticals, Nürnberg, Germany) and laser photocoagulation merely slowed the progression of visual acuity loss. [6-8]

Although intravitreal injections of ranibizumab have been shown to be effective in the treatment of neovascular AMD, not all patients demonstrated improvements in visual acuity, and some had lost ≥3 lines of VA by the end of the MARINA and ANCHOR trials [1,2].

In this study performed in a clinical setting, 35% of the patients were considered to be reduced responders to treatment at the end of follow-up, having either reduced visual acuity compared to baseline and/or persistent intraretinal or subretinal fluid or persistent or recurrent choroidal neovascularisation.

In a cohort receiving as-needed injections of ranibizumab for exudative AMD, visual improvement was related to the frequency of injections received but not to the resolution of fluid by OCT [9]. Ahlers and co-workers identified subretinal fluid as the most relevant factor for visual function [10].

Brown et al. stated that if a monthly reinjection protocol is not used and patients are treated on an as-needed basis, a combination of clinical examination and qualitative OCT measurements should be used to guide the anti-VEGF treatment to maximise vision gain [11]. The importance of closely monitoring patients is confirmed by our study, as 35% of the patients presented a reduction in visual acuity compared to baseline and/or persistent intraretinal or subretinal fluid or persistent or recurrent choroidal neovascularisation at the end of follow-up.

Menghini et al. retrospectively evaluated predictive factors for being a “good” or “bad” responder to ranibizumab treatment [12]. They found that only the course of visual acuity in the first three months seems to be of value for estimating the treatment response; however, they identified no predictor for response to treatment, e.g. lesion size [12].

Rosenfeld and co-workers investigated the cause of visual acuity (VA) loss in patients with neovascular age-related macular degeneration (AMD) receiving monthly ranibizumab injections in pivotal ranibizumab phase-III trials. At month 24, 9% of the ranibizumab-treated patients from MARINA and 10% of the ranibizumab-treated patients from ANCHOR had lost >/=15 letters VA. Baseline characteristics associated with VA loss at month 24 included older age, better VA, and larger lesions [13].

Surprisingly, unlike other authors, we did not find a correlation between age and a reduced response to treatment [13,14]. However, other investigators have also not found a correlation between age and response to treatment [15]. Our findings should be verified in a larger population with a longer follow-up.

Defining the proportion of reduced responders in the MARINA and ANCHOR trials is not entirely possible, as both report the proportion of patients improving by more than 15 letters, and the primary end point was the proportion of patients losing fewer than 15 letters [1,2]. The proportions of patients improving by more than 15 letters were 33.8% for the 0.5 mg group in the MARINA study and 40.3% for the 0.5 mg group in the ANCHOR study [1,2]. In our study, only one patient (0.6%) lost three or more lines of vision at the end of follow-up; conversely, 99.4% of patients lost fewer than three lines. In the MARINA and ANCHOR trials, this proportion was 94.6% in the 0.5 mg group in the MARINA and 96.4% in the 0.5 mg group in the ANCHOR study [1,2]. In our study, we observed only 17.6% of patients with improvement by more than 3 lines, but one must consider that we included all CNV types, including those with RAP lesions and the 12.1% of patients with a pigment epithelium detachment at baseline. Thus, the results are difficult to compare. Reche-Frutos et al. stated that RAP II lesions with PED and RAP III have an poorer anatomic and visual evolution than patients with stage II without PED after ranibizumab therapy [16]. Another difference is that we had a follow-up of six months, whereas the MARINA and ANCHOR studies had 12 months of follow-up.

Muether and co-workers found a correlation between the time elapsed between treatment indication and first injection and the visual acuity deterioration [17]. Other authors reported a correlation between visual acuity deterioration in 28.4% of patients and a median treatment delay of 28 days [18]. In our study, the time elapsed between the diagnosis and the first ranibizumab treatment was not a predictor of a reduced response to treatment, according to the criteria listed above. In Germany, payment for the initial ranibizumab treatment and PRN treatment must be pre-approved by the patient’s public health insurance company. In Germany, the predominantly visual acuity-driven ranibizumab retreatment regimen is based on the EMA (European Medicines Agency) drug information, which recommends retreatment after recurrent vision loss of 5 EDTRS letters. Second, lesion activity (persistent or recurrent subretinal fluid, increase of pigment epithelium detachment, new haemorrhage, recurrent thickening of the retina >100 μm) was recommended as an important criterion for retreatment by the German Ophthalmological Society (DOG), the Professional Association of German Ophthalmologists (BVA) and the Retinological Society (RG).

Because we defined reduced responders as patients with either decreasing visual acuity at the end of follow-up or a reduced anatomical response to treatment, those patients who experienced a delay in the onset of therapy may have already had further alterations in the retinal pigment epithelium or fibrosis. However, in our study, visual acuity at baseline was no predictor of a reduced response to treatment.

Our study is limited by its retrospective nature, and a further weakness is the limited follow-up of six months. In Germany, reimbursement for the OCT is not covered by public health insurance. Therefore, as the patient has to pay for the OCT, it was not performed every four weeks. Moreover, the present study did not differentiate the extent of fibrosis as a predictor for reduced response to treatment. The presence of active choroidal neovascularisation was an inclusion criterion for enrolment in the study; thus, further studies investigating the presence and extent of fibrosis in the analysis of predictors are needed. Another limitation of our present study is its short follow-up, as large lesions in this short time period might not have been sufficiently treated. Furthermore, large initial lesion sizes might simply reflect a more severe nature of the disease than smaller lesion sizes, rather than a reduced response to treatment. Further studies with a longer follow-up will be necessary to verify whether lesion size is truly a predictor of reduced response.

The “reduced responder” poses challenges to clinicians, and there is no general consensus on how “reduced responders” are defined. The existing studies are very heterogeneous, and comparisons with published studies are difficult. The definition of a “reduced responder” in our study was chosen as stated above in order to include patients with decreasing visual acuity at the end of follow-up, as well as those showing a reduced anatomical response to treatment.

## Conclusions

In this study performed in a clinical setting, we have identified initial lesion size as a predictor for a poor treatment response. It remains difficult to predict patient response prior to treatment. Intravitreal anti-VEGF treatment for large lesions is absolutely essential, as untreated, these lesions have the potential to cause severe vision loss and fibrosis. However, patients with a large initial lesion size should be thoroughly informed about the potentially poorer response to the intravitreal treatment.

## Competing interests

The authors declare that they have no competing interests.

## Authors’ contributions

All of the authors contributed substantially to this study, the analysis and interpretation of the clinical data, participated in development and writing of the manuscript, and approved the final draft for publication.

## Pre-publication history

The pre-publication history for this paper can be accessed here:

http://www.biomedcentral.com/1471-2415/13/84/prepub
